# Long-term effect of yogic practices on diurnal metabolic rates of healthy subjects

**DOI:** 10.4103/0973-6131.36761

**Published:** 2008

**Authors:** M S Chaya, H R Nagendra

**Affiliations:** Department of Life Sciences, Vivekananda Yoga Anusandhana Samsthana (VYASA), Bangalore, India; 1 Vyasa (Swami Vivekananda Yoga University), Prasanti Kuteeram, Jigani, Bangalore Dist-560 019, India

**Keywords:** Basal Metabolic Rate (BMR), decreased arousal response, yoga, yoga training

## Abstract

**Background:**

The metabolic rate is an indicator of autonomic activity. Reduced sympathetic arousal probably resulting in hypometabolic states has been reported in several yogic studies.

**Aim:**

The main objective of this study was to assess the effect of yoga training on diurnal metabolic rates in yoga practitioners at two different times of the day (at 6 a.m. and 9 p.m.).

**Materials and Methods:**

Eighty eight healthy volunteers were selected and their metabolic rates assessed at 6 a.m. and 9 p.m. using an indirect calorimeter at a yoga school in Bangalore, India. Results and conclusions: The results show that the average metabolic rate of the yoga group was 12% lower than that of the non-yoga group (*P* < 0.001) measured at 9 p.m. and 16% lower at 6 a.m. (*P* < 0.001). The 9 p.m. metabolic rates of the yoga group were almost equal to their predicted basal metabolic rates (BMRs) whereas the metabolic rate was significantly higher than the predicted BMR for the non-yoga group. The 6 a.m. metabolic rate was comparable to their predicted BMR in the non-yoga group whereas it was much lower in the yoga group (*P* < 0.001). The lower metabolic rates in the yoga group at 6 a.m. and 9 p.m. may be due to coping strategies for day-to-day stress, decreased sympathetic nervous system activity and probably, a stable autonomic nervous system response (to different stressors) achieved due to training in yoga.

An understanding of metabolism at different times of the day has significant implications due to its link to sleep, health, stress and fatigue, ultimately determining the quality of life. BMR is the lowest (basal) metabolic rate measured just after waking from a night's sleep.[1] It is well known that sleep is a restorative process wherein the body synthesizes its necessary chemicals for growth, maintenance and development. Rejuvenation and repair take place during sleep.[2] Sleep generally brings down the metabolic rate by about 10%.[1,3] Low basal metabolic rates of yoga practitioners have been reported.[4] Hypometabolic states have been reported in yogic studies[5–8] wherein short and immediate effects of meditation and relaxation were observed.

Yoga which is a way of life is characterized by balance, health, harmony and bliss.[9] In yoga lore, three normal states of consciousness have been identified[10]—they are wakeful state (*jagratha*), dream or rapid eye movement (REM, *swapna* or dream state) and deep sleep without dreams (*sushupti*). Meditation is a part of yoga which is the seventh limb of *Astanga* yoga[11]—a state of alert rest as stated by Maharshi Mahesh Yogi.[12] This leads to superconsciousness or the fourth state of consciousness called the Samadhi, which is beyond the deep sleep state in terms of metabolism.[5,6]

Practice of yoga brings about changes such as slowing of the breath, calming of the mind and relaxation of the body leading to efficiency/balance or homeostasis.[9] The earliest study on metabolism by Anand *et al.*[13] has demonstrated that a yogi could reduce oxygen consumption while sealed in an airtight box for nearly ten hours, thus changing metabolism at will. Studies on meditation have demonstrated that Transcendental meditation (TM),[5,6] Zen Meditation,[8] Om Meditation[7] and Yogic Relaxation[14] reduce oxygen consumption, respiratory and heart rates and spontaneous Galvanic Skin Response (GSR).[15,16] The above changes are attributed mainly to reduced sympathetic activity / arousal response and decreased mental and muscular activities.

Studies done on a certain type of Pranayama (breathing technique) called UJJAYI demonstrated a 19% increase in oxygen consumption.[17] Right nostril breathing increased oxygen consumption by 28%.[18] Practice of Asanas or specific postures also increased oxygen consumption.[19,20] All these changes have been seen as the immediate acute effect of yoga practices. There is no literature report as to how long these changes last or the influence these practices have on the diurnal metabolic rates measured at two different times of the day in experienced yoga practitioners.

A typical yoga capsule usually consists of Asana, Pranayama and deep relaxation or meditation, which has a combined effect but ultimately leads to deep relaxation and reduced oxygen consumption.[4] It is believed that all these must have some permanent effect on metabolism when practised over a period of time, leading to efficiency and relief from stress, thereby resulting in certain changes in the diurnal metabolism, BMR and sleep.

Wallace *et al.* have reported the effect of acute practices resulting in the reduction in metabolic rate in meditation as compared to sleep.[5,6] The present study examines whether there is also a long-term effect of the combined practices.

With this background, the present study was conducted to know: (1) Is there any difference in the diurnal metabolic rate at two different times of the day (6 a.m. and 9 p.m.) of trained yoga subjects compared to those in the non-yoga group? (2) To compare the metabolic rate at 6 a.m. with that of the subjects' BMR values predicted by the 1985 Food and Agricultural Organization/World Health Organization/United Nations University (FAO/WHO/UNU) equations.[21]

## SUBJECTS AND METHODS

The study was conducted at VYASA, a residential yoga research foundation near Bangalore. Out of 140 subjects screened, 88 (39 women, 49 men) normal, healthy subjects (as assessed by clinical examination) were selected for the study. These subjects were not taking any medication and did not have any sleep disorder. They were in the age range of 20–55 years (mean age = 33 yrs ± 9.88) with a mean weight of 58.36 ± 8.94 kg, mean height of 1.48 meters ± 0.23 and a mean body mass index (BMI) of 21.59 ± 3.09). The yoga group consisted of 27 men and 24 women who were trained in yoga for a minimum period of six months and who had been practising yoga for at least two hours a day, five days a week for 25 days a month (Appendix A). The non-yoga control group consisted of 22 men and 15 women who did not practice yoga but were staying in the same yoga institute under similar environmental conditions performing day-to-day activities of the center. Both the groups had similar activity profiles (other than yoga) and took the same simple vegetarian meal. The two groups were matched for weight, age and BMI [Table 1].

**Table 1:** Age and anthropometrical characteristics of men and women of Yoga and Non-Yoga groups

Groups		Total	Men	Women
	*n*	51	27	24
Yoga	Age (y)^ns^	33.44± 10.17	33.96±9.1	32.92±11.24
	Weight (kg)^ns^	58.23± 8.53	60.59±8.89	55.88±8.18
	Height (m)^ns^	1.49±3.4	1.55±0.23	1.43±0.21
	BMI (kg/m^2^)^ns^	21.61±3.40	21.02±3.03	22.21±3.78
	*n*	37	22	15
Non-Yoga	Age (y)^ns^	33.75± 9.59	34.18±9.65	33.33±9.53
	Weight (kg)^ns^	58.5±9.34	61.73±9.43	55.27±9.26
	Height (m)^ns^	1.48± 0.24	1.55±0.25	1.42±0.24
	BMI (kg/m^2^)^ns^	21.58±2.78	21.12±2.77	22.04±2.79

Values are mean ± 1 standard deviation.

ns: Non-Significant between groups (Independent t test.)

m: Meters, BMI: Body mass index

All subjects had an early, simple, non-spicy vegetarian dinner at 6 p.m. The assessments were made early in the morning at around 6 a.m. and between 8.30 and 9 p.m. to eliminate the thermic effect of food on metabolism. The subjects were randomized for assessment, 50% of them being assessed at 9 p.m. and the other 50% at 6 a.m. Assessments were made in the morning only after the subjects had reported that they had slept for about seven hours.

The subjects were made to rest in a supine position for 20–30 minutes before the assessments were made twice for 15 min each with a gap of 15 minutes. The measurements were made in thermoneutral conditions at approximately 25°C in quiet surroundings and the average of two readings was taken.

The assessments were done by indirect calorimetry using OXYCON PRO from JAEGER, Germany. The instrument was calibrated daily for flow volume and gas analysis by using certified gases (mixture of 5.2% CO_2_ in nitrogen and atmospheric air, BOC, UK). VO_2_ (volume of oxygen), VCO_2_ (volume of carbon dioxide), VE (Ventilation), BF (Breath Flow), EE (Energy Expenditure) and RQ (Respiratory Quotient) were measured by triple V sensors-fast response gas analyzer based on the differential paramagnetic principle using a face mask. The gas analyzer is designed to analyze gases with a speed of 10 msec (100 Hz) with a breathing level of up to 80 breaths per minute.

The 1985 FAO/WHO/UNU equations (26) were used to predict values of BMR based on age, sex and body weight.

### Statistical analysis

The data was analyzed using independent t test for comparing the yoga and non-yoga groups, paired t test for analyzing the intragroup effects. Analysis of co-variance was used to adjust the BMR for differences in body weights between the groups. SPSS 10 package was used for all the analyses. Differences were considered significant at *P* < 0.05 for all statistical procedures. The data is presented as mean ± standard deviation (SD).

## RESULTS

Table 2 gives the details of energy expenditure of the yoga and non-yoga groups and their predicted values of two different assessments taken at 6 a.m. and 9 p.m. The yoga group showed significantly lower metabolic rates both at 6 a.m. and at 9 p.m. when compared to the non-yoga group (*P* < 0.001, *P* < 0.05). The 9 p.m. measurements of the yoga group were almost equal to their predicted values (before sleep). On the other hand, the non-yoga group had significantly higher metabolic rates before sleep and basal metabolic rates (SMR and BMR) comparable to their predicted values at 6 a.m. The EE/kg body weight values were also significantly lower in the yoga group at 6 a.m. and 9 p.m. (*P* < 0.01) when compared to the non-yoga group. The yoga group had significantly lowered values in other measured parameters as well such as VO_2_ (*P* < 0.03), VCO_2_ (*P* < 0.05), heart rate (*P* < 0.01) at 9 pm when compared with the non-yoga group and had lower values in VO_2_ (*p* < 0.001), VCO_2_ (*p* < 0.05), Breath rate (*p* < 0.05).

**Table 2:** Energy Expenditure of Yoga and Non-yoga groups at 9 p.m. and 6 a.m.

Metabolic rate at two different times of the day	Yoga *n* = 51	Non-Yoga *n* = 37
9 p.m. MR (Kcal.d^-1^)	1413.79±402.2*	1604.17±326.86
6 a.m. MR (Kcal.d^-1^)	1200.57±242.41**‡	1415.17±278.22
6 a.m. MR (Kcal.d-1.kg^-1^)	20.61±4.16*‡	24.19±4.76
Predicted BMR (Kcal.d^-1^)	1385.36±165.24	1399.51±159.94
Predicted BMR (Kcal.d-1.kg^-1^)	23.79±2.84	23.92±2.73

Values are expressed as mean ± 1 SD.

MR: Metabolic Rate, BMR: basal metabolic rate

2. Significant at *P* < 0.05 comparisons between yoga and non-yoga groups, independent t test

** Significant at *P*< 0.001 using 9 p.m. values as covariate (Ancova), adjusted for body weight.

‡ Significant at *P* < 0.001 comparisons between yoga and non-yoga groups.

Independent † test compared with measured energy expenditure using predicted values.

ns Not Significant

Gender differences in energy expenditure and VO_2_ continued even after adjusting for body weight in the yoga group whereas no gender differences existed in the non-yoga group after adjusting for body weight (Ancova).

## DISCUSSIONS

The main result of this study is that the yoga group had significantly lower metabolic rates at two different times of the day, i.e., 6 a.m. and 9 p.m. when compared to the non-yoga group. The yoga training had a beneficial effect on metabolism in general as seen in the lowered metabolic rate of the yoga group at 9 p.m. and reduced EE/kg body weight suggesting greater metabolic efficiency.

An earlier study by Anand *et al.*[13] on a yogi shut in an airtight box for ten hours showed reduced metabolic rate at the end of this period. However, the basal metabolic rate of the yogi had been within the normal range.

Wallace *et al.*[5,6] has shown that the practice of transcendental meditation leads to hypometabolic states (lowered metabolic rates) and proposed to call it the fourth state of consciousness, different from sleep yet metabolically equivalent or even below metabolic rates seen during sleep. He observed a 17% reduction in oxygen consumption during 20 min of transcendental meditation whereas after sleep, the metabolic rate was usually reduced by 10%.[2,3] Our study demonstrates that the acute effects of yoga practiced over a period of time, will have long-term effects on metabolism, leading to metabolic efficiency (as seen in the lowered EE/kg body weight).

High-intensity exercises increase the basal metabolic rates in subjects aged between 59–77[22] years whereas low and moderate intensity exercises do not have any effect on these rates.[23,24] All these exercises are mainly related to central or sympathetic activation[25] whereas yoga is reported to bring down sympathetic activity.[5,8,14] However, Yoga Asana (physical posture) is an energy expenditure activity[19,20] and hence, it was hypothesized that it would increase the resting metabolic rate. Contrary to this hypothesis, our study shows that Asanas when practised along with pranayama and meditation over a period of time actually reduce the metabolic rate. This may be due to alterations in autonomic nervous functions leading to autonomic stability as observed by Orme-Johnson,[15] who found that those practising TM showed stability in the rate of habituation, number of multiple responses and spontaneous GSR when compared to non-meditators. Silverman *et al.*[16] also observed low levels of sympathetic activity which have been correlated with greater resistance to stresses such as sensory deprivation. This deprivation can be attributed to decreased arousal response (reduced sympathetic activation) after TM meditation.

**Table 3:** Genderwise comparison of BMR of Yoga and Non-Yoga groups along with their predicted values

	Yoga		Non-Yoga	

Metabolic rates at two different times of the day	Women *n* = 24	Men *n* = 15	Women *n* = 27	Men *n* = 22
9 p.m. MR (Kcal.d^-1^)	1141.71±299.87^ns^	1639.78±265.4§	1358.16±340.02^ns^	1781.51±234.35
9 p.m. (Kcal.d-1.kg^-1^)	20.75±5.47	24.32±4.8	27.06±5.61	28.86±3.8
6 a.m. MR (Kcal.d^-1^)	1045.57±201.9**‡	1324.62±226.16**‡	1283.90±207.32^ns^	1510.67±273.92^ns^
6 a.m. MR (Kcal.d^-1^.kg^-1^)	18.99±3.61**‡	22.07±4.09*‡	23.37±3.42	24.76±4.44
Predicted BMR Kcal.d^-1^)	1242.14±93.36	1505.96±108.77	1256.06±90.92	1504.9±103.24

Values are in mean ± 1 SD

MR = Metabolic rate

** Significant at *P* < 0.005 comparisons between yoga and non-yoga groups

2. Significant at *P* < 0.05 comparisons between yoga and non-yoga groups, multivariate analysis using 9 p.m. values as covariate

‡ Significant at *P* < 0.001 compared to predicted values

ns: Not Significant when compared with predicted values.

**Table 4:** Genderwise comparison of yoga and non-yoga groups in all parameters other than energy expenditure

	Yoga		Non-Yoga	

Ventilatory parameter and heart rate	Women *n* = 24	Men *n* = 15	Women *n* = 27	Men *n* = 22
9 p.m. VE [l/min]	6.15±1.64	7.96±1.83	6.56±1.55	8.53±1.11
6 a.m. VE [l/min]	5.35±1.03^ns^	6.17±1.00**	6.22±1.25	7.4±1.46
9 p.m. BF [1/min]	17.52±10.54	14.44±4.57	19.50±2.38	14.8±4.13
6 a.m. BF [1/min]	14.49±3.52*	12.43±3.72**	17.86±2.14	14.38±3.09
9 p.m. VO_2_, [ml/min]	166.15±38.83	237.44±45.17	195.99±37.74	253.28±33.65
6 a.m. VO_2_ [ml/min]	153.35±26.23**	195.46±27.62*	186.47±31.93	216.29±38.8
9 p.m. VCO_2_ [ml/min]	154.07±38.22	220.00±47.09	174.52±31.86	238.16±30.5
6 am VCO_2_ [ml/min]	140.36±25.08*	173.21 ±26.92**	161.81 ±26.41	198.87±36.31
9 pm HR [beats/min]	69.06±9.32	68.16±9.06	78.63±8.56	72.62±8.29
6 am HR [beats/min]	69.64±8.99^ns^	65.09±9.9^ns^	72.09±5.76	67.84±12.45

Values mean ± SD

** Significant at *P* <0.005 genderwise comparison between yoga and non-yoga groups, multivariate analysis using 9 p.m. values as covariate.

2. Significant at *P* < 0.05 genderwise comparison between yoga and non-yoga groups, multivariate analysis using 9 p.m. values as covariate

ns: Not Significant, VE: Ventilation, BF: Breath flow, VO_2_: Volume of oxygen

VCO_2_: Volume of carbon dioxide, HR: Heart rate.

There are other yoga techniques such as Om meditation,[7] Zen meditation,[8] Cyclic meditation[14] and certain pranayamas (breathing techniques),[17,26] in which decreased metabolic rate, heart rate, reduction in Oxygen consumption, carbon dioxide elimination, breath rate, breath volume and pulse rate were observed as acute effects (pre- compared to post- levels). Electroencephalograms (EEGs) recorded by Banquet *et al.*[27] showed predominant alpha wave activities with increased amplitude and frequency but few theta waves during the practice of Transcendental meditation. Our study not only endorses this view but also adds that the integrated approach of yoga training can convert these short-term changes into more permanent expressions as noticed in the changes in metabolic rates of the yoga group at 6 a.m. and 9 p.m. and the changes in EE/kg body weight.

The BMR which includes the cost of arousal, represents the activation of the central and sympathetic nervous systems from sleep to basal levels[28] and may be influenced by various factors including control over the state of one's mind.[9]

The lower 6 a.m. metabolic rate of the yoga group suggests that the combined practice of yoga has an overall calming effect and is manifested as decreased sympathetic activity.[6,7,14] Orme Johnson[15] has further demonstrated that greater the regularity of practice, greater was the increase in autonomic stability. This is also amply demonstrated by the lowered metabolic rates of the yoga group both at 6 a.m. and 9 p.m. compared to the metabolic rates of the non-yoga group in our study. These lower metabolic rates can be attributed to the decreased sympathetic tone and decreased arousal response in general and a greater ability to recover from stressful conditions quickly. This is evident when we look at the differences in the yoga and non-yoga groups at 9 p.m., which existed even after adjusting for body weight (using Ancova).

Furthermore, studies on the long-term effects of yoga on the BMR of normal healthy volunteers have shown that yoga groups had lower BMR compared to non-yoga groups[4] which has also been confirmed by our study.

There are however, contradictory reports about gender differences in SMR/BMR, a few claiming that the differences do not exist when adjusted for Body weight, Fat mass or Fat-Free Mass[28–30] while others claim that gender differences exist even after accounting for these parameters.[31,32] In our study, gender differences although not found in the non-yoga group, existed in the yoga group. This suggests that training in yoga may have different effects on men and women as women respond differently to stress.[33,34] A further detailed study on the effect of training on gender differences along with a detailed analysis of body composition (Fat mass, Fat-free mass and Fat distribution) may be necessary to determine the precise cause of these differences.

We conclude that the long-term practice of yoga leads to lower metabolic rates and probably greater metabolic efficiency mainly due to reduced sympathetic activity and / or stabilized nervous system. The main limitation of this study is that it is not a prospective randomized control study and hence, conclusions can not be drawn based on these results. However, the results are consistent with expectations based on other studies and thus, encouraging to conduct further investigations.

## APPENDIX A

### Details of yoga practices done by the yoga group selected for the study

All the subjects practise a 60–90 min capsule consisting of Asana, Pranayama and Meditation daily for six days a week.

### Breathing

1. Hands in and out
2. Ankle stretch
3. Tiger
4. Rabbit

### Loosening

1. Heals touching
2. Tadasana techniques

### Asana Practices: Standing posture

1. Trikona asana
2. Parivrutha trikonasana
3. Ardha kati Chakrasana
4. Ardha Chakrasana

### Sitting Asanas

1. Paschimothanasan
2. Ushtrasana
3. Vakrasna/Matyendrasana

### Supine postures

1. Halasana
2. Chakrasana
3. Sarvangasana
4. Matsyasana

### Prone postures

1. Bhujanghasana
2. Shalabhasana
3. Dhanurasana

### Pranayama

1. Kapala Bhathi.
2. Nadi shuddi
3. Omkar chanting
